# Incentive and Reminder Strategies to Improve Response Rate for Internet-Based Physician Surveys: A Randomized Experiment

**DOI:** 10.2196/jmir.6318

**Published:** 2016-09-16

**Authors:** David A Cook, Christopher M Wittich, Wendlyn L Daniels, Colin P West, Ann M Harris, Timothy J Beebe

**Affiliations:** ^1^ Mayo Clinic Online Learning Mayo Clinic College of Medicine Rochester, MN United States; ^2^ Knowledge Delivery Center Mayo Clinic College of Medicine Rochester, MN United States; ^3^ Division of General Internal Medicine Mayo Clinic Rochester, MN United States; ^4^ Survey Research Center Mayo Clinic Rochester, MN United States; ^5^ Division of Biomedical Statistics and Informatics Mayo Clinic Rochester, MN United States

**Keywords:** surveys and questionnaires, survey methods, questionnaire design

## Abstract

**Background:**

Most research on how to enhance response rates in physician surveys has been done using paper surveys. Uncertainties remain regarding how to enhance response rates in Internet-based surveys.

**Objective:**

To evaluate the impact of a low-cost nonmonetary incentive and paper mail reminders (formal letter and postcard) on response rates in Internet-based physician surveys.

**Methods:**

We executed a factorial-design randomized experiment while conducting a nationally representative Internet-based physician survey. We invited 3966 physicians (randomly selected from a commercial database of all licensed US physicians) via email to complete an Internet-based survey. We used 2 randomly assigned email messages: one message offered a book upon survey completion, whereas the other did not mention the book but was otherwise identical. All nonrespondents received several email reminders. Some physicians were further assigned at random to receive 1 reminder via paper mail (either a postcard or a letter) or no paper reminder. The primary outcome of this study was the survey response rate.

**Results:**

Of the 3966 physicians who were invited, 451 (11.4%) responded to at least one survey question and 336 (8.5%) completed the entire survey. Of those who were offered a book, 345/2973 (11.6%) responded compared with 106/993 (10.7%) who were not offered a book (odds ratio 1.10, 95% CI 0.87-1.38, *P*=.42). Regarding the paper mail reminder, 168/1572 (10.7%) letter recipients, 148/1561 (9.5%) postcard recipients, and 69/767 (9.0%) email-only recipients responded (*P*=.35). The response rate for those receiving letters or postcards was similar (odds ratio 1.14, 95% CI 0.91-1.44, *P*=.26).

**Conclusions:**

Offering a modest nonmonetary incentive and sending a paper reminder did not improve survey response rate. Further research on how to enhance response rates in Internet-based physician surveys is needed.

## Introduction

Surveys remain a widely used, cost-effective means of assessing the attitudes, beliefs, and practices of physicians. However, conducting physician surveys is challenging. Response rate, a benchmark commonly used in appraising survey quality, is typically 10% lower in physician surveys than in nonphysician surveys [1,2] and appears to be decreasing for many physician subgroups [3,4]. Although a high response rate may not be the best predictor of overall survey quality [5,6], low response rates raise concerns about study precision, nonresponse bias, and the overall inferential value of study findings.

Recent reviews of the literature on physician survey response behavior have identified the method of initial contact, mode of administration, and approach to nonrespondent follow-up as important determinants of response rate [4,7-10]. Electronic survey methods, and Internet-based surveys in particular, are becoming increasingly popular owing to their potential for reduced time spent in collecting data, immediate availability of data once collected, improved data quality, and overall cost savings [11-16]. However, electronic modalities often have lower response rates than paper mailed surveys [4,7,11,15-24].

Although uncertainties remain regarding how to enhance physician response rate in Internet-based surveys, incentives constitute one possible support. Incentives increase the perception of value, trust, reciprocity, and appreciation on the part of the respondent [4,25]. Research indicates that incentives improve response rates and that monetary incentives are more effective than nonmonetary incentives [3,7,8,26,27], yet most studies investigating the impact of incentives on physician survey response have done so in the context of mailed, paper-based forms [11]. There remains a paucity of evidence concerning how incentives work in the context of electronic surveys of physicians.

Physician survey response can also be improved through multiple follow-up contacts, although the optimum number of contact attempts remains unclear [27]. In the context of an Internet-based survey, sending follow-up reminders via email to invitees who fail to complete an Internet-based survey after the initial invitation costs virtually nothing [11]. However, repeated email reminders may backfire, hardening prospective respondents’ resistance to future requests. Thus, email reminders may be less desirable than reminders via other methods (eg, mail or telephone). In one study, even after sending the nonresponding physicians 4 emailed reminders, the overall response rate improved substantially after investigators sent nonresponding physicians a paper letter with a printed URL that the physicians used to access the Internet-based survey [12]. Little consensus exists regarding what reminder approaches are most efficient and effective in increasing response rates among physicians, regardless of data collection mode. This investigation is a response to specific calls for further testing in this area [28,29].

Despite the importance of physician surveys and ongoing concerns over participation, few randomized trials have examined potential strategies to address nonresponse [7,25]. Moreover, even when physician surveys are optimally executed (and enjoy high levels of participation) the investigators infrequently describe the challenges they encountered [3,28]. As such, significant gaps remain in our understanding of best practices in physician survey research. We sought to address these knowledge gaps.

We hypothesized that a low-cost nonmonetary incentive and a paper mail reminder would improve response rates in an Internet-based survey of physicians. We also hypothesized that a personalized formal letter would have only a slightly greater benefit than a personalized postcard. We tested these hypotheses using a factorial-design randomized experiment in the context of a nationally representative Internet-based physician survey. To our knowledge, no one has applied such a design to study the process of conducting physician surveys.

## Methods

### Survey Procedures

From September to December 2015, we conducted a self-administered Internet survey among a random sample of licensed physicians in the United States. The survey sought physician opinions regarding 2 topics that we believed they would perceive as personally important, namely, maintenance of certification and continuing medical education. Maintenance of certification is presently a matter of controversy and debate [30,31], and both this and continuing medical education [32,33] directly affect the professional lives of nearly all physicians. Details of survey development and results will be published separately.

The survey was administered using Qualtrics (Qualtrics LLC, Provo, UT, USA). All invitees received an email (Textbox 1) inviting them to complete the survey, accessible via an embedded URL link unique to each invitee. Consistent with the protocol used by Braithwaite and colleagues [15] in which it took 5 reminder emails to achieve a 52% response rate, we sent 6 reminder emails on days 11, 20, 31, 36, 48, and 58. A subset of participants also received 1 paper reminder as outlined below.

**Initial invitation email.**

[Email subject line: CME and MOC – Survey of physicians' needs]

Dear Dr. [last name],

Continuous professional development (CPD), including maintenance of certification (MOC), affects all physicians. Our group is trying to promote changes to make CPD easier and more effective.

We invite you to participate in a nation-wide survey, and make your voice heard on these important issues! *Those who complete the survey can request a free copy of the “Mayo Clinic Handbook for Happiness: A Four-Step Plan for Resilient Living.”*

This survey asks physicians' opinions about MOC, continuing medical education, and online learning. We want to understand what you do to maintain your professional knowledge and skills, what challenges you face, and—most importantly—what needs to change.

We anticipate this will take about 7 minutes to complete. Your responses will be anonymous. We would be grateful to receive responses by October 21, 2015.

Click here to start the survey.

We thank you in advance for your participation. Feel free to contact any of us if you have questions.

Sincerely,

[study investigators]

This study has been approved by the Mayo Clinic Institutional Review Board. All responses will be anonymous (unless otherwise explicitly noted) and strictly confidential.

-------------------------

Editorial note: The italicized text was deleted in the “no book offer” group for the initial email and all subsequent reminders. The email text varied slightly for each follow-up email, but the incentive text remained unchanged. CME = continuing medical education.

### Interventions

**Figure 1:**
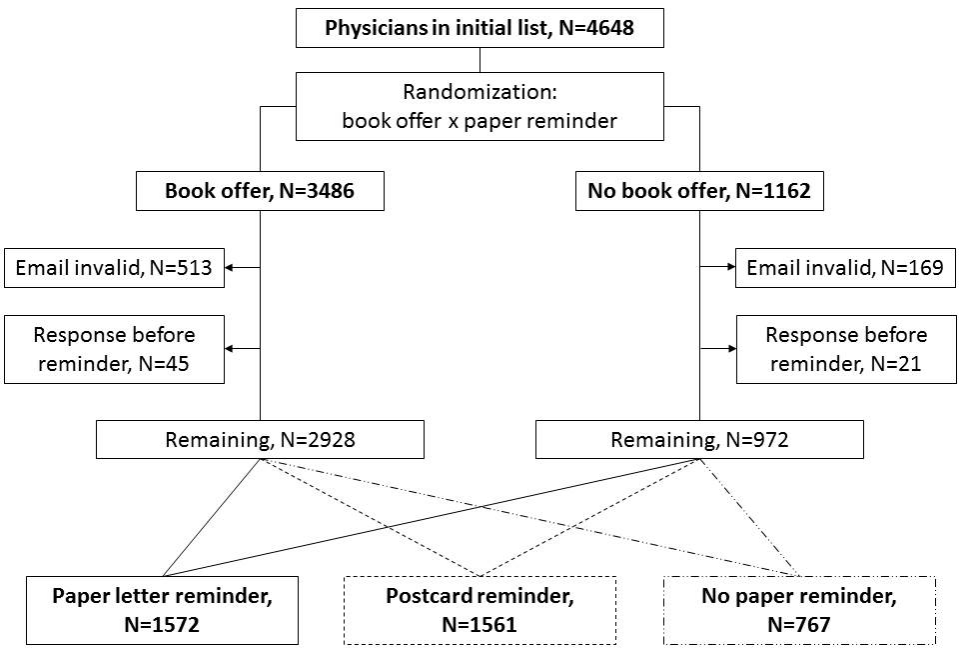
Participant flow.

This factorial-design study tested 2 interventions (Figure 1). First, we systematically altered the emails inviting physicians to participate, with one version offering a book (the *Mayo Clinic Handbook for Happiness: A Four-Step Plan for Resilient Living* nominal cost about US $10) upon completion of the survey and the other version making no mention of any tangible incentive (Textbox 1). Although the wording of the reminder emails varied slightly, the presence or absence of the book offer remained constant. We randomly assigned 75% of invitees to receive emails with the book offer. All respondents were offered the book upon completion of the survey; the intervention in this case was the *up-front notification* regarding the book.

Second, 7 days after the first email we sent a reminder via paper mail to a subset of invitees using two formats. We timed this mailing to arrive at approximately the same time as the first email reminder. The invitees in one group received a personalized letter (Multimedia Appendix 1, Box 1), printed on institution letterhead bonded paper and sealed in an envelope, asking them to complete the survey using the link they had received via email, or to contact the study investigators if they had not received or had deleted the email. A second group received a similar message via a personalized postcard (Multimedia Appendix 1, Box 2) that included the institution logo on both sides and was signed by one of the investigators. A third group received no paper reminder. We randomly assigned 40% of the initial sample to the letter group, 40% to the postcard group, and 20% to the no paper reminder group. Only 1 paper reminder was sent.

### Human Subjects

We obtained a sample of 4648 names and email addresses selected at random from the LexisNexis Provider Data Management and Services database of all licensed US physicians (LexisNexis Risk Solutions, Alpharetta, GA). This study was approved by the Mayo Clinic Institutional Review Board. Survey response was tracked but responses were deidentified before analysis. Randomization for both interventions was done at the same time before the survey began by a research assistant using a random number generator (Microsoft Excel).

### Outcomes and Data Collection

The primary outcome of this study was the survey response calculated using the American Association for Public Opinion Research formula RR2 (Response Rate 2) [34], that is, the number answering at least one survey question divided by the number of surveys sent less those returned as undeliverable. Secondary outcomes included the number of respondents who completed the entire survey, those who actually claimed a book, and time to response (defined as the number of days between the initial survey mailing and the response date). We obtained demographic information on physician sex, age, degree, specialty, and practice location from the LexisNexis database. For analysis purposes we dichotomized physician specialty as generalist (family medicine, general internal medicine, or general pediatrics) and nongeneralist.

### Data Analysis

We compared response rates between interventions and across physician subgroups using the chi-squared test and report odds ratios with 95% confidence intervals for dichotomous variables. We used logistic regression to explore potential interactions between the interventions and physician demographic subgroups. We graphed time to response to visually explore its association with reminder emails. We performed statistical analyses using SAS version 9.4 (SAS Institute, Inc, Cary, NC, USA), using two-sided tests and *P* ≤ .05 as the threshold of statistical significance. To achieve 80% power to detect an absolute improvement of 5%, we estimated we would need to invite 4000 physicians (providing 81% power assuming baseline response rate of 10%, 87% power assuming baseline rate of 25%). All participants were analyzed in the groups to which they were originally assigned (“intent to treat”).

## Results

### Survey Response

We obtained the email addresses of 4648 licensed physicians, randomly assigned these to intervention arms (Figure 1), and sent them an email invitation. We received notification that 682 emails were undeliverable, leaving 3966 potential respondents (Table 1). Of these 3966 physicians, 451 (11.4%) responded to at least one survey question during the study period and 336 (8.5%) completed the entire survey. Twenty-six physicians clicked the survey link but did not answer any questions, and 84 opted out of the survey. We received no response during the study period for the remaining 3405 emails.

Of the undeliverable emails, for 673 we received notice that the email address was invalid and for 9 we received automated messages indicating that the physician would only accept emails from approved senders. One representative message read, “To control spam, I now allow incoming messages only from senders I have approved beforehand. If you would like to be added to my list of approved senders, please fill out the short request form (see link below).” We did not fill out such forms.

### Impact of Book Incentive Offer

**Figure 2:**
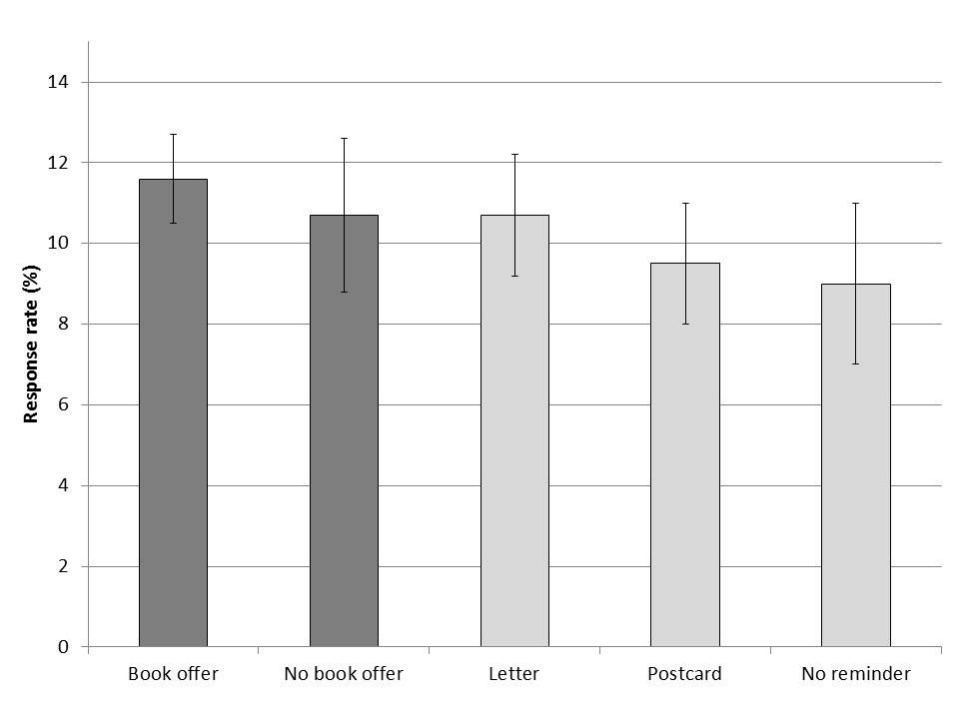
Survey response rate with different incentives and reminders. Dark gray bars show response rate by incentive (book offer or none), *P*=.42. Light gray bars show response rate by paper reminder (letter, postcard, or none), *P*=.35. Error bars indicate the 95% confidence interval for the binomial proportion.

Of 2973 physicians who were offered a book in the email invitation, 345 (11.6%) responded to at least one survey question compared with 106 of 993 (10.7%) physicians who were not offered a book (odds ratio 1.10, 95% CI 0.87-1.38, *P*=.42); see Figure 2. Converting this odds ratio to absolute rates relative to the baseline rate for those not offered a book, the 95% CI for response among those offered a book ranged from 9.5% to 14.2%.

Among the 336 respondents who completed the entire survey, 224 (66.7%) requested a book. Those who had been offered a book ultimately requested one slightly more often (177/257, 68.9%) than those who had not been offered a book (47/79, 59.5%; odds ratio 1.51, 95% CI 0.89-2.54, *P*=.12).

**Table 1:** Demographics of invitees and respondents.

Demographic feature		Invitees (N=3966)^a^	Respondents (N=451)^a^
Age in years, mean (SD; interquartile range)		54.9 (11.5; 46-63)	55.3 (10.5; 47-62)
Male, n (%)		2637 (66.5)	290 (64.3)
Degree, MD^b^, n (%)		3791 (95.6)	442 (98.0)
**Specialty**^c^, n (%)			
	General medicine or pediatrics	1244 (31.5)	117 (25.9)
	Surgery or anesthesia	1041 (26.4)	128 (28.4)
	Nongeneral clinical	1412 (35.8)	174 (38.6)
	Diagnostic	253 (6.4)	32 (7.1)
**Region**^d^, n (%)			
	Northeast	852 (21.5)	98 (21.7)
	Midwest	803 (20.3)	97 (21.5)
	South	1364 (34.4)	151 (33.5)
	West	941 (23.8)	105 (23.3)

^a^ Invitees are physicians who were sent an email that was not returned as undeliverable; respondents are those who answered one or more survey questions. Responses may not sum to column total N owing to missing data.

^b^ MD: doctor of medicine. Non-MD degrees were doctor of osteopathy (DO).

^c^ General medicine includes family medicine and internal medicine; surgery includes ophthalmology and obstetrics/gynecology; nongeneral clinical includes medical subspecialties (cardiology, pulmonology, etc), dermatology, emergency medicine, neurology, and physical medicine, among others; diagnostic includes radiology and pathology and their subspecialties.

^d^ Regions are classified according to US Census Bureau definitions.

### Impact of Paper Reminders

Sixty-six physicians responded before receiving the mailed paper reminders. Among the remaining 3900 physicians, the mailed paper reminders had no significant impact on response rates, with 168/1572 (10.7%) letter recipients, 148/1561 (9.5%) postcard recipients, and 69/767 (9.0%) email-only recipients responding to at least one survey question (*P*=.35). When dichotomized as any paper reminder (316/3133, 10.1%) versus none, the results were again not statistically significant (odds ratio 1.13, 95% CI 0.86-1.49, *P*=.36). Converting this odds ratio to absolute rates relative to the baseline rate for those not receiving a paper reminder, the 95% CI for response among those receiving any paper reminder ranged from 7.9% to 12.8%. Comparison of the response rate for those receiving letters or postcards was likewise not statistically significant (odds ratio 1.14, 95% CI 0.91-1.44, *P*=.26).

Results for both the book incentive and paper reminder were similar when only those who completed the entire survey were counted as respondents (data not shown).

### Associations of Response Rate With Demographic Features

We explored associations between response rate and several demographic features. Response rate varied significantly by age (*P*<.001), with both younger (response rate 8.8% for age < 45 years) and older (9.0% for age ≥65 years) physicians responding less frequently than others (12.8% for age group 45-54 years, 14.9% for age group 55-64 years). We found no significant difference in response rates across specialties as classified in Table 1 (*P*=.06); however, generalist (family medicine, internal medicine, and pediatric) physicians responded less often (9.4%) than nongeneralist physicians (12.3%; *P*=.007). Physicians with a doctor of medicine (MD) degree were twice as likely (442/3791, 11.7%) to respond as those with a doctor of osteopathy (DO) degree (9/174, 5.2%, *P*=.008). We found no significant difference by sex (*P*=.30) or geographic region (*P*=.90).

In multivariate analysis simultaneously accounting for all 5 demographic variables, namely, age, specialty (generalist vs nongeneralist), degree, sex, and region, statistically significant predictors of response were age (*P*<.001), specialty (*P*=.003), and sex (161/1329, 12.1% female, 290/2637, 11.0% male; *P*=.02).

### Interaction Between Interventions and Demographic Features

We explored interactions between the study interventions and demographic features as well. Independent adjustment for age, specialty (generalist or nongeneralist), sex, and geographic region did not change the finding of no effect from the book incentive or the reminder. We found no significant interaction between any intervention and any of these demographic features (*P*_interaction_ ≥.09).

### Influence of Email Reminders

**Figure 3:**
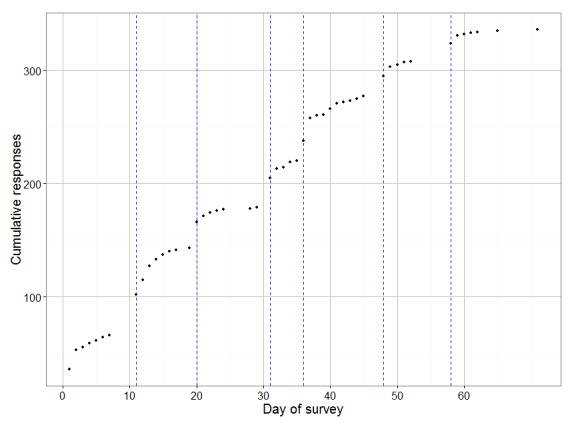
Timeline of responses for completed surveys.The survey started on day 1. Vertical dashed lines indicate the dates of reminder emails. The paper reminder was mailed on day 7, with the intent that it would arrive on the same day as the first reminder email. Intervals with no dots indicate periods in which no additional responses were received.

Figure 3 shows a timeline of cumulative responses and illustrates the continued favorable impact of repeated reminder emails.

## Discussion

### Summary of Findings

Optimizing the response rate of Internet-based surveys is a matter of great importance to survey researchers. In this randomized experiment, we found that offering a modest incentive had no impact on response rate in an Internet-based survey. We also found that paper reminders had no impact on response rate, whether in the form of a formal letter or a postcard. Generalist (family medicine, internal medicine, and pediatric) physicians responded less often than nongeneralist physicians, and middle-aged physicians responded more often than younger or older physicians, but we found no significant interaction between the study interventions and specialty or age. We observed a meaningful number of additional responses after each of the 6 reminder emails.

### Limitations

The greatest limitation of this study is the overall low response rate. A recent meta-analysis [4] estimated the average survey response rate among health professionals at 53%; however, a review focused on Internet surveys of physicians found that response rates <20% are not uncommon [11]. Although the low response rate does not affect the internal validity of the study findings, it could affect the generalizability of these results to other survey contexts. We note that despite the low response rate, the confidence intervals exclude what would be considered by most researchers to be a meaningful difference in response rate.

One possible explanation for the overall low response rate is that email addresses were invalid or not monitored, but we were not notified. If true, this would not affect our conclusions unless there were a systematic bias in the distribution of invalid emails (which seems unlikely). Another possibility is that physicians perceived relatively low value in this incentive—perhaps they simply were not interested in this particular book. Research suggests that an unappealing incentive in some situations may be worse than no incentive at all. For example, one study found that offering free continuing medical education credit to Internet survey respondents actually resulted in lower response rates compared with no incentive [35]. Alternatively, the promise that the book would be delivered at a later date might have been less motivating than an immediately present, tangible incentive. The logistical difficulty of providing an immediate meaningful incentive reflects a limitation of Internet-based surveys generally [36,37] and among physicians specifically [11]. A third possibility is that the invitees did not find the survey topic interesting. Like other professionals, physicians will not complete a survey if the topical importance is unclear or perceived as low [25]. Although maintenance of certification and continuing medical education appear prominently in current physician discussion venues, it is possible that the invited physicians perceived low personal salience in these topics.

Strengths of our study include the randomized design and adequate power to detect even small intervention effects, if present.

### Integration With Prior Work

Prior investigations of methods for physician surveys, conducted primarily in the context of paper surveys, indicate that incentives increase participation. Our findings suggest that in Internet-based surveys the incentive may play a smaller role, perhaps because the promise of a future reward prompts less motivation than would a tangible gift. This suggestion is supported by prior research [38-40] indicating that noncontingent prepaid monetary incentives (eg, including US $5 with an initial paper survey invitation) yield higher response rates than contingent postcompletion offers, such as that used in our investigation.

Research also suggests that sending paper reminders after an initial email invitation or offering the opportunity to complete the survey via the Internet or on paper increases physician survey response rates [19,41]. We hoped that a single paper reminder might have a similar effect, but unfortunately, such was not the case.

The field of electronic surveys continues to evolve. Tools to conduct such surveys have become more ubiquitous, more powerful, and more user-friendly in recent years. While these improvements make it easier for health service researchers to conduct Internet-based survey research, it also makes it easier for everyone else to do the same. Thus, physicians (along with many other people) often feel overwhelmed by survey invitations (“surveyed to death”) and other unsolicited emails (“spam”) and may indiscriminately ignore, delete, or opt out of all survey requests. In addition, the increasing prevalence and awareness of electronic threats (eg, computer viruses, phishing, and identity theft) lead people to protect themselves from emails arising from unknown senders. A small percentage of the physicians invited to participate in our survey blocked all emails from unknown senders, a solution that may become more popular in coming years. The evolving landscape of Internet-based surveys will continue to challenge those using this mode of administration.

### Conclusions and Implications for Research Methods

Our study has several implications for best practices in survey research. First, the book incentive did not improve response rates. Because this contradicts prior work on incentives in paper surveys, it remains to be seen whether this finding was simply related to the particular incentive or survey topic or whether it indicates a general lack of benefit from contingent nonmonetary incentives in the context of Internet-based physician surveys.

Second, the paper reminder also did not help, whether in the form of a postcard or a formal letter. Assuming this finding replicates in future work, it suggests that researchers using electronic surveys will need to find other ways to encourage response. Mixed-modality surveys using both paper and Internet will likely achieve the best results.

Third, repeated email reminders continued to work, at least through 6 reminders spaced at approximately 10-day intervals. The long-term impact of such reminders (eg, potential negative impact on physicians' response to other surveys) could not be evaluated in this study.

The methods of Internet-based survey research continue to evolve. What was once a powerful and easily accessible tool may no longer continue to be such, given the current state of electronic threats and excessive surveys from marketing, customer satisfaction, and political pollsters as well as health care service researchers. Extra effort may be required to follow other approaches known to improve survey response, such as highlighting the salience of the topic, having someone known to the invitee champion the survey, broadening the options for survey completion to include additional modes (eg, fax, telephone, interactive voice response telephone, or face-to-face), and decreasing the burden of survey completion by utilizing the briefest forms possible.

## Appendix

Multimedia Appendix 1 Details of paper reminder letters.
