# Supplementary material for: Incentive and Reminder Strategies to Improve Response Rate for Internet-Based Physician Surveys: A Randomized Experiment
Source: J Med Internet Res. 2016 Sep 16;18(9):e244. doi: 10.2196/jmir.6318 (PMC5045523; doi:10.2196/jmir.6318)
Supplement: Multimedia Appendix 1 [file jmir_v18i9e244_app1.pdf]

SUPPLEMENTAL DIGITAL CONTENT

Online Box 1. Paper reminder letter[a]

[Mayo Clinic logo]

[recipient name, address]

Dear Dr. [last name],

Continuous professional development (CPD), including maintenance of certification (MOC), affects all physicians. Our group is trying to promote changes to make CPD easier and more effective.

We invite you to participate in a nation-wide survey, and make your voice heard on these important issues! ***Those who complete the survey can request a free copy of the "Mayo Clinic Handbook for Happiness: A Four-Step Plan for Resilient Living."***

This survey asks physicians' opinions about MOC, continuing medical education, and online learning. We want to understand what you do to maintain your professional knowledge and skills, what challenges you face, and – most importantly – what needs to change.

Last week we sent you an email with a link to the survey. If you have already responded to this survey, thank you very much; you may disregard this reminder.

If not, then please use that link to access the survey. If you did not receive this email, but would like to participate, please contact <email address of study investigator>.

We anticipate this will take about 7 minutes to complete. Your responses will be de-identified and strictly confidential. We would be grateful to receive responses by <date 2 weeks out>, although responses after that date will still be useful.

Thanks in advance for your participation. Feel free to contact any of us if you have questions.

Sincerely,

<signature>

[names of study investigators]

[a] The bolded, underlined text was deleted in the "no book offer" group. The letter was printed on institution letterhead bonded paper and mailed in a sealed envelope exhibiting the Mayo Clinic logo.

Online Box 2. Postcard reminder[a]

[Mayo Clinic logo]

Dear Dr. [last name],
Continuous professional development (CPD), including maintenance of certification (MOC), affects all physicians. Our group is trying to promote changes to make CPD easier and more effective.

As part of this effort we are conducting a nationwide survey of physicians' opinions about MOC, continuing medical education (CME), and online learning. We invite you to participate in this survey, and make your voice heard! (If you have already responded thank you very much; you may disregard this reminder.)

Last week we sent you an email with a link to the survey. Please use that link to complete the survey. If you did not receive this email, but would like to participate, please contact <email address of study investigator>.

Those who complete the survey can request a free copy of the "Mayo Clinic Handbook for Happiness: A Four-Step Plan for Resilient Living."

Feel free to contact any of us if you have questions.

Thanks in advance!

<signature>

[names of study investigators]

[a] The bolded, underlined text was deleted in the "no book offer" group. Distinguishing features of the postcard included a watermark image of the Mayo Clinic founders (the Mayo brothers) as a background to the message, and the Mayo Clinic logo printed on both sides of the postcard.

References <move>

1. Asch DA, Jedrziewski MK, Christakis NA. Response rates to mail surveys published in medical journals. J Clin Epidemiol 1997;50:1129-1136.
2. Asch S, Connor SE, Hamilton EG, et al. Problems in recruiting community-based physicians for health services research. J Gen Intern Med 2000;15:591-599.
3. McLeod CC, Klabunde CN, Willis GB, et al. Health care provider surveys in the United States, 2000-2010: a review. Eval Health Prof 2013;36:106-126.
4. Cho YI, Johnson TP, Vangeest JB. Enhancing surveys of health care professionals: a meta-analysis of techniques to improve response. Eval Health Prof 2013;36:382-407.
5. Davern M. Nonresponse rates are a problematic indicator of nonresponse bias in survey research. Health Serv Res 2013;48:905-912.
6. Johnson TP, Wislar JS. Response rates and nonresponse errors in surveys. JAMA 2012;307:1805-1806.
7. VanGeest JB, Johnson TP, Welch VL. Methodologies for improving response rates in surveys of physicians: a systematic review. Eval Health Prof 2007;30:303-321.
8. Kellerman SE, Herold J. Physician response to surveys. A review of the literature. Am J Prev Med 2001;20:61-67.
9. Thorpe C, Ryan B, McLean SL, et al. How to obtain excellent response rates when surveying physicians. Fam Pract 2009;26:65-68.
10. Klabunde CN, Willis GB, Casalino LP. Facilitators and barriers to survey participation by physicians: a call to action for researchers. Eval Health Prof 2013;36:279-295.
11. Dykema J, Jones NR, Piche T, et al. Surveying clinicians by web: current issues in design and administration. Eval Health Prof 2013;36:352-381.
12. Dykema J, Stevenson J, Day B, et al. Effects of incentives and prenotification on response rates and costs in a national web survey of physicians. Eval Health Prof 2011;34:434-447.
13. Minniear TD, McIntosh EB, Alexander N, et al. Using electronic surveys to gather information on physician practices during a response to a local epidemic--Rhode Island, 2011. Ann Epidemiol 2013;23:521-523.
14. Schleyer TK, Forrest JL. Methods for the design and administration of web-based surveys. J Am Med Inform Assoc 2000;7:416-425.
15. Braithwaite D, Emery J, De Lusignan S, et al. Using the Internet to conduct surveys of health professionals: a valid alternative? Fam Pract 2003;20:545-551.
16. Akl EA, Maroun N, Klocke RA, et al. Electronic mail was not better than postal mail for surveying residents and faculty. J Clin Epidemiol 2005;58:425-429.
17. Crouch S, Robinson P, Pitts M. A comparison of general practitioner response rates to electronic and postal surveys in the setting of the National STI Prevention Program. Aust N Z J Public Health 2011;35:187-189.
18. Leece P, Bhandari M, Sprague S, et al. Internet versus mailed questionnaires: a controlled comparison. J Med Internet Res 2004;6:e39.
19. McMahon SR, Iwamoto M, Massoudi MS, et al. Comparison of e-mail, fax, and postal surveys of pediatricians. Pediatrics 2003;111:e299-303.
20. Nicholls K, Chapman K, Shaw T, et al. Enhancing response rates in physician surveys: the limited utility of electronic options. Health Serv Res 2011;46:1675-1682.
21. Raziano DB, Jayadevappa R, Valenzula D, et al. E-mail versus conventional postal mail survey of geriatric chiefs. Gerontologist 2001;41:799-804.
22. Seguin R, Godwin M, MacDonald S, et al. E-mail or snail mail? Randomized controlled trial on which works better for surveys. Can Fam Physician 2004;50:414-419.
23. Thomas M, Rogers R, Maclean R. Collecting data from physicians via web-based surveys: recommendations for improving response rates. Intern J Med Inform 2003;1:ce9.
24. VanDenKerkhof EG, Parlow JL, Goldstein DH, et al. In Canada, anesthesiologists are less likely to respond to an electronic, compared to a paper questionnaire. Can J Anaesth 2004;51:449-454.
25. VanGeest JB, Beebe TJ, Johnson TP. Surveys of Physicians. In: Johnson TP, ed. Handbook of Survey Methods. Hoboken, NJ: John Wiley & Sons, Inc; 2014:515-543.

26. Burns KE, Duffett M, Kho ME, et al. A guide for the design and conduct of self-administered surveys of clinicians. CMAJ 2008;179:245-252.
27. Field TS, Cadoret CA, Brown ML, et al. Surveying physicians: do components of the "Total Design Approach" to optimizing survey response rates apply to physicians? Med Care 2002;40:596-605.
28. Klabunde CN, Willis GB, McLeod CC, et al. Improving the quality of surveys of physicians and medical groups: a research agenda. Eval Health Prof 2012;35:477-506.
29. Willis GB, Smith T, Lee HJ. Do additional recontacts to increase response rate improve physician survey data quality? Med Care 2013;51:945-948.
30. Iglehart JK, Baron RB. Ensuring physicians' competence--is maintenance of certification the answer? N Engl J Med 2012;367:2543-2549.
31. Cook DA, Holmboe ES, Sorensen KJ, et al. Getting maintenance of certification to work: a grounded theory study of physicians' perceptions. JAMA Intern Med 2015;175:35-42.
32. Nissen SE. Reforming the Continuing Medical Education System. JAMA 2015;313:1813-1814.
33. Institute of Medicine. Redesigning Continuing Education in the Health Professions. Washington, DC: The National Academies Press; 2010.
34. Viera AJ, Edwards T. Does an offer for a free on-line continuing medical education (CME) activity increase physician survey response rate? A randomized trial. BMC Res Notes 2012;5:129.
35. Göritz AS. Using lotteries, loyalty points, and other incentives to increase participant response and completion. In: Gosling SD, Johnson JA, eds. Advanced methods for conducting online behavioral research. Washington, DC: American Psychological Association; 2010:219-233.
36. Göritz AS. Incentives in Web Studies: Methodological Issues and a Review. International Journal of Internet Science 2006;1:58-70.
37. James KM, Ziegenfuss JY, Tilburt JC, et al. Getting physicians to respond: the impact of incentive type and timing on physician survey response rates. Health Serv Res 2011;46(1 Pt 1):232-242.
38. Kanaan RA, Wessely SC, Armstrong D. Differential effects of pre and post-payment on neurologists' response rates to a postal survey. BMC Neurol 2010;10:100.
39. Leung GM, Johnston JM, Saing H, et al. Prepayment was superior to postpayment cash incentives in a randomized postal survey among physicians. J Clin Epidemiol 2004;57:777-784.
40. Beebe TJ, Locke GR, 3rd, Barnes SA, et al. Mixing web and mail methods in a survey of physicians. Health Serv Res 2007;42:1219-1234.
